# Median cleft lip: A new method of surgical repair

**DOI:** 10.4103/0970-0358.63965

**Published:** 2010

**Authors:** B. V. Khandekar, S. Srinivasan, N. J. Mokal

**Affiliations:** Burns and Plastic Surgery Department, B.J. Wadia Hospital, Parel, Mumbai, India

**Keywords:** Midline cleft lip, muscle repair

## Abstract

The aim is to discuss a new method of muscle repair in midline cleft lip. Three patients with midline cleft lip were repaired with our technique of muscle repair and the results evaluated. Our new method of muscle repair in the form of 'Z' helps in forming the philtral dimple.

## INTRODUCTION

Median or midline cleft lip is defined as any congenital vertical cleft through the centre of the lip.

It can occur as a sporadic event, or as a part of an inherited sequence of anomalies. It arises due to incomplete merging of the median nasal prominences which form the inter-maxillary segment.

Two major categories of midline cleft lip are described:

1. Demyer sequence:[1,2] Frontonasal deformity associated with hypotelorism, holoprosencephaly and facial deformity which ranges from cyclopia to midline facial cleft with pre-maxillary agenesis.
2. Median Cleft face syndrome: It is often associated with nasal deformity, hypertelorism usually either with no or little brain deformity (corpus callosum agenesis). Amongst these patients surgical reconstruction is feasible due to the probability of normal life expectancy.

## CASE REPORT

Three cases of median cleft lip were repaired with our new method of muscle repair.

### Surgical technique

Surgery was planned after anaesthesia work-up. Methylene Blue was used to mark the philtral column to allow the construction of a philtral base of 8 mm and a columellar base of 6 mm.

**Figure 1:**
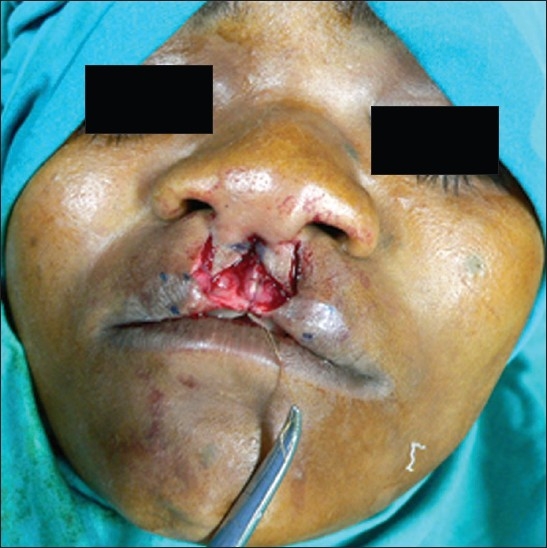
Forked flaps for columella

Forked flaps were marked on the edges of the cleft to construct the columella [Figure 1].[3]

Simple paring of the edges in an inverted 'V' fashion was done in one case to construct the columella.

**Figure 2:**
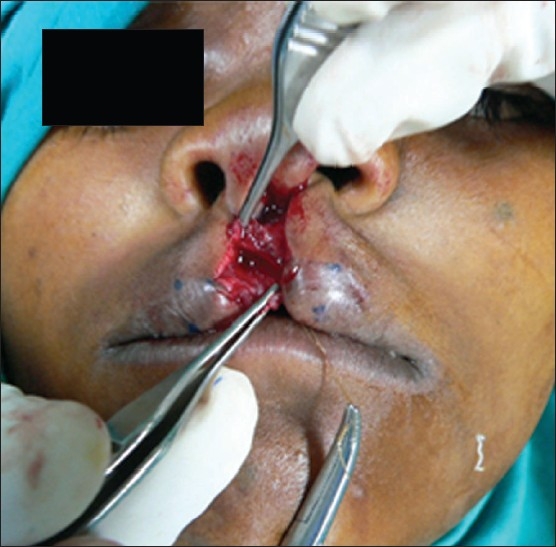
Muscle repair in "Z" form

The orbicularis oris was dissected from the skin and mucosa. The abnormal muscular insertions of the nostril sill and alar margins were released. However, the suturing of the muscle was done in the form of "Z" instead of transverse repair [Figure 2]. Muscle was closed with vicryl 4-0.

**Figure 3:**
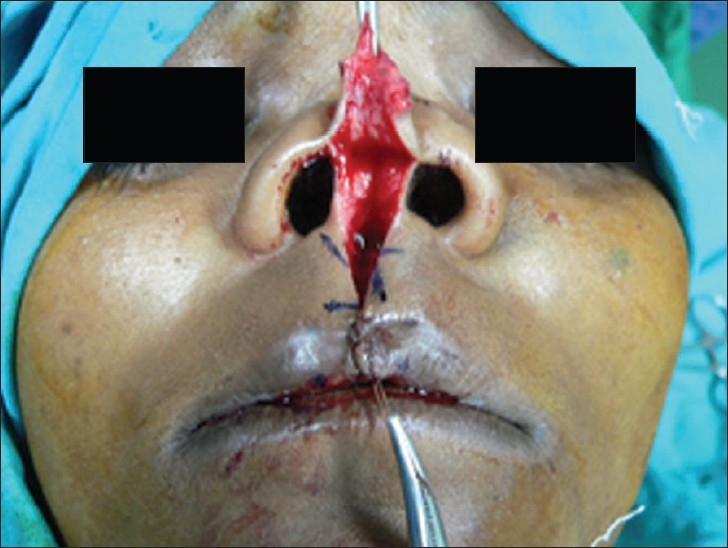
Markings of triangular flaps

**Figure 4:**
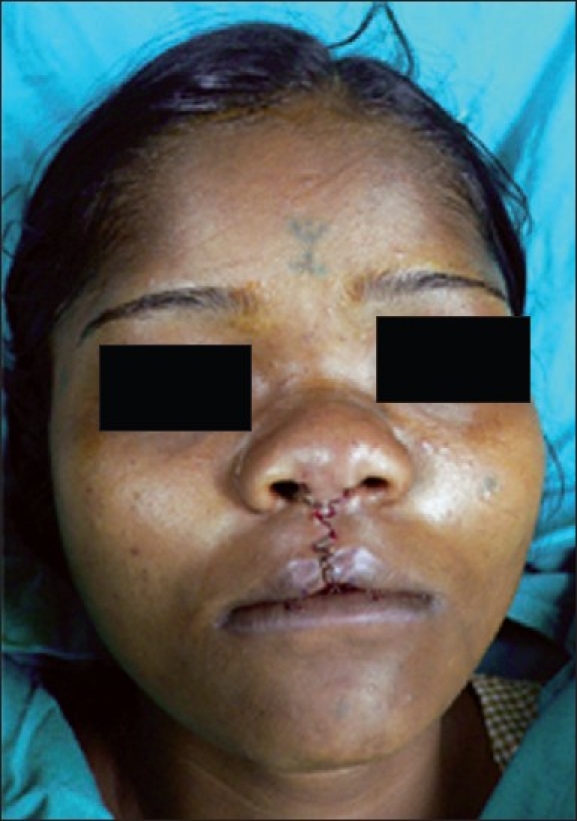
Immediate post-op

Two triangular flaps were marked on the skin [Figure 3] and skin closure was completed with ethilon 6-0 [Figure 4].

**Figure 5:**
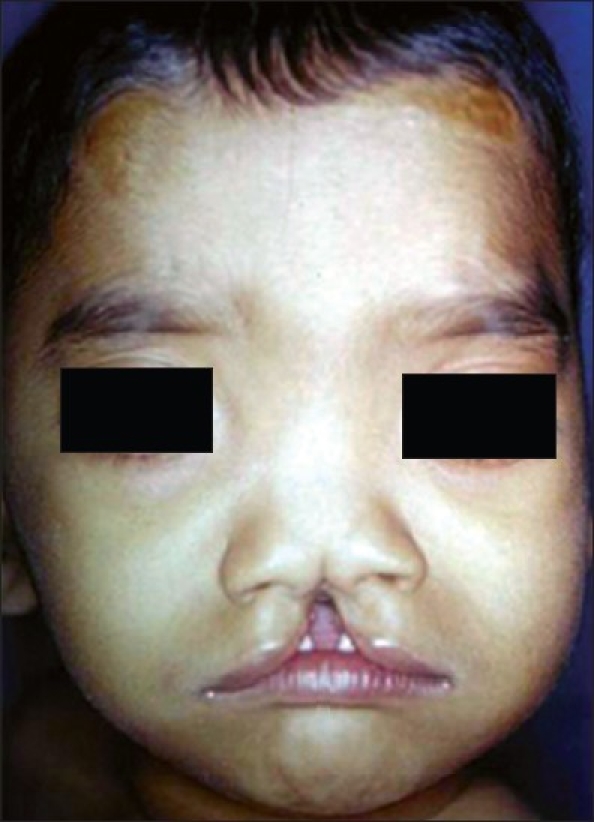
Pre-op photograph (Case 2)

**Figure 6:**
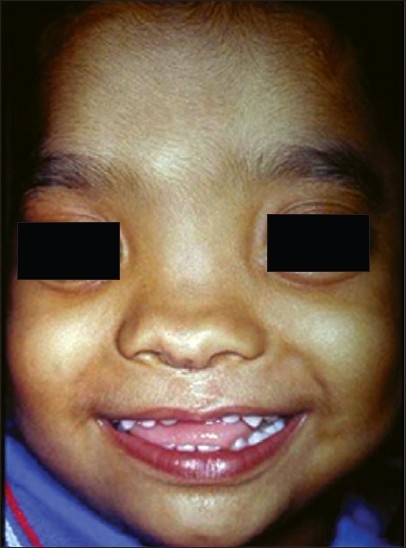
Post-op photograph (Case 2)

Sutures were removed on the seventh postoperative day. The similar technique was used in case no. 2 [Figure 5] with late follow-up showing [Figure 6] philtral dimple.

## DISCUSSION

Median or midline cleft lip is a rare anomaly which occurs with a incidence of 0.43 to 0.73% in cleft population.[4]

Surgical correction is feasible, since intelligence levels and life expectancy are normal in these cases.

In the literature greater importance is given to pathogenesis than to surgical repair. In our surgical technique we have addressed all the elements of median cleft lip deformity like columellar length, nostril width, philtral height and appearance, to achieve a well-balanced lip.

Forked flaps are used to reconstruct the columella which is most often deficient in these cases. Particular attention is given to the abnormally inserted orbicularis oris, which was released from the alar base and nostril sill, which helped in reducing the nostril width. This method of muscle repair in the form of a 'Z' predominantly helped in the formation of the philtral dimple.

The philtral height is achieved and maintained with two triangular flaps,[4] as described earlier, which avoids straight line closure. Considering all the features of median cleft deformity during surgical correction and using a different method of muscle repair in the form of 'Z' it was possible to achieve a functionally and aesthetically acceptable lip.
